# MicroRNA-144 suppresses cholangiocarcinoma cell proliferation and invasion through targeting platelet activating factor acetylhydrolase isoform 1b

**DOI:** 10.1186/1471-2407-14-917

**Published:** 2014-12-05

**Authors:** Rui Yang, Yongjun Chen, Cong Tang, Hongbo Li, Bing Wang, Qun Yan, Junbo Hu, Shengquan Zou

**Affiliations:** Department of General Surgery, Affiliated Tongji Hospital, Tongji Medical College of Huazhong University of Science and Technology, 1095 Jiefang Avenue, Wuhan, Hubei 430030 China; Department of General Surgery, The Fifth Affiliated Hospital of Sun Yat-sen University, Zhuhai, Guangdong 519000 China; Department of General Surgery, The Fifth Hospital of Wuhan, Wuhan, Hubei 430037 China

**Keywords:** CCA, miRNA, Cell proliferation, Cell invasion, LIS1

## Abstract

**Background:**

MicroRNAs are endogenous non-coding RNAs that play important roles in a wide variety of biological processes such as apoptosis, development, aging and cancer. The aberrant expression of miRNAs may contribute to phenotypic features of malignant cells, including resistance to chemotherapy. However, in cholangiocarcinoma (CCA) the correlation between miRNAs and their potential roles in CCA remains unclear.

**Methods:**

MicroRNA profiles were analyzed in three pairs of CCA tumor specimens and non-tumorous-paired biliary tissues using Agilent microRNA microarrays. Expression of selected miRNAs was further confirmed in CCA tissues and CCA cell lines by q-PCR. The effects of miR-144 were evaluated by cell proliferation, migration, transwell, and tumorigenicity assays. Expression of LIS1 (platelet-activating factor acetylhydrolase isoform 1b) was assessed in CCA specimens and CCA cell lines by q-PCR and western blot. Targeting of LIS1 by miR-144 was confirmed by luciferase reporter assays.

**Results:**

We found that the expression of 28 miRNAs in CCA tissues was significantly different from their corresponding adjacent normal bile duct tissues. We focused on miR-144 which was significantly down-regulated in CCA tissues. Reintroduction of miR-144 in CCA cell lines not only inhibited cell growth, but also significantly reduced cell migration and invasion capacities compared with controls. Luciferase assays and western blots verified LIS1 as a direct target of miR-144, and knocking-down LIS1 has similar effect with overexpression of miR-144 in CCA cell lines. Moreover, overexpression of miR-144 expression could suppress tumor growth in nude mice.

**Conclusions:**

Our results showed that miR-144 was reduced in CCA tissues and suggested that miR-144 may be an essential suppresser of CCA cell proliferation and invasion through targeting LIS1.

**Electronic supplementary material:**

The online version of this article (doi:10.1186/1471-2407-14-917) contains supplementary material, which is available to authorized users.

## Background

Cholangiocarcinoma (CCA) is a malignant tumor of bile duct epithelial cells, and the incidence and prevalence of CCA have been increasing worldwide over recent decades [1, 2]. The survival rate of patients with CCA is very poor with a median survival of 6–12 months, as it is most often diagnosed at an advanced stage with intrahepatic and/or lymph node metastases [3–5]. Despite advances in surgical techniques, systemic chemotherapy and/or radiotherapy, radical surgery remains the only curative treatment for this devastating disease [6–10]. Therefore, an improved understanding of the molecular mechanisms of tumor initiation, progression, and metastasis formation of CCA is urgently required as the basis to identify novel therapeutic targets and develop effective therapeutic strategies.

MicroRNAs (miRNAs) are endogenous, single-stranded, non-coding, small RNAs that regulate gene expression by preferentially binding to specific sequences in the 3′-untranslated region (3′-UTR) of their target mRNAs [11]. Accumulating evidence indicates that aberrant expression of miRNAs contributes to a variety of biological processes including embryonic development and tumorigenesis [12–15]. Several studies have demonstrated significant changes of miRNA expression levels in CCA tissue in comparison with paired noncancerous bile duct. Furthermore, deregulated miRNAs have been identified that act as oncogenes or tumor suppressors [16–22]. These results suggest that miRNAs can contribute to tumor growth, although the possible molecular mechanisms remain to be further elucidated.

In the present study, we first performed a comprehensive analysis of miRNA expression profiles in CCA tissues and paired noncancerous bile ducts. We found that miR-144 was significantly down-regulated in CCA tissues and CCA cell lines. To investigate the role of miR-144 in cancer cells, we examined the cellular effects of miR-144 overexpression. We also identified LIS1 as a novel target gene of miR-144. Furthermore, LIS1 silencing could imitate the phenomenon of miR-144 overexpression. These results provide insight into the molecular mechanisms of CCA and may offer a novel therapeutic target in this disease.

## Methods

### Tissue samples

A total of 70 paired human CCA samples with histological evidence were obtained from the Department of Biliary-Pancreatic Surgery, Affiliated Tongji Hospital (Hubei, China). Tumor tissues and the corresponding adjacent normal tissues were frozen in liquid nitrogen and stored at -80°C until use. Written informed consent was obtained from all patients and the study was approved by the Institutional Review Boards of the Affiliated Tongji Hospital of Huazhong University of Science and Technology.

### MiRNA microarrays and miRNA target prediction

Total RNA was extracted from three CCA tissues and matched adjacent, non-tumor bile duct tissues using TRIZOL (Invitrogen, Carlsbad, CA, USA) according to the supplier’s instructions. The miRNA expression profile was determined using the Agilent Human miRNA Microarray Kit (V2) (Agilent Inc., Santa Clara, CA, USA) (Sanger database v.12.0). Hybridized microarray slides were scanned with the Agilent Scanner G2565A and Agilent Feature Extraction version 9.5 was used to extract signals. Data analyses including Gene Ontology analysis, Pathway Analysis, and MicroRNA-gene network were performed (shbiochip, Shanghai, China). The target genes of miR-144 were obtained from public databases (miRanda, PicTar, and Target ScanS) according to the following two criteria: the target gene contained the conserved 8-mer and 7-mer sites that match the seed region of miR-144, and the target gene was predicted by at least two programs.

### Cell culture and transient transfection

Human embryonic kidney HEK293T cells were obtained from American Type Culture Collection, and human CCA cell lines (HCCC-9810, CCLP1, HuCC-T1, and RBE) were conserved by our laboratory.The nonmalignant cholangiocyte cell line BECwere generously provided by Hiromi Ishibashi, Japan.All cells were maintained in DMEM or RPMI 1640 supplemented with 10% v/v fetal bovine serum (FBS) (Gibco, Grand Island, NY, USA), 100 U/mL penicillin, and 100 μg/mL streptomycin at 37°C in a humidified incubator containing 5% CO_2_.

For transfection, HuCC-T1 and RBE cells were transfected with anti-miR-144 inhibitors (anti-miR-144) or Anti-miRNA Inhibitors Negative Control #1 (NC) (Applied Biosystems, Foster City, CA, USA) in a final concentration of 20 nM using X-tremeGENEsiRNA Transfection Reagent (Roche, Indianapolis, IN, USA), according to the manufacturer’s instructions. The expression levels of miR-144 were quantified 24 h after transfection, and the cells were examined by western blot analysis 48 h after transfection. HCCC-9810 and CCLP1 cells were transfected with specific chemosynthesized siRNA sequences for targeting human LIS1 (gene ID: 5048) or NC. The sequences of siRNA are supplied in Additional file 1: Table S1. Silencing of LIS1 was confirmed by western blot analysis 48 h after transfection.

### Plasmid constructs and lentiviral transduction

To generate the lentiviral vector pCDH-miR-144 that overexpresses miR-144, a fragment encoding the pre-miR-144 sequence was amplified by PCR from HEK293T cell genomic DNA and then cloned into the BamHI/EcoRI sites of the pCDH-CMV-EF1-copGFP vector (SBI, Mountain View, CA, USA).

The sequences of the LIS1 3′-UTR containing putative seed sequences of miR-144 were amplified from CCLP1 genomic DNA and cloned into the psiCHECK™-2 vector (Promega, Madison, WI, USA). The cloned 3′-UTR of LIS1 was mutated using Quickchange (Stratagene, San Diego, CA, USA). The sequences of primer are supplied in Additional file 1: Table S1. The successful sequences and insertions were confirmed by DNA sequencing.

For production of viral particles, we co-transfected the lentivirus-mediated miR-144 packaging system containing pCDH-CMV-EF1-copGFP or PCDH-miR-144, Rec, TAT, Gag, and Vsvg into HEK293T cells with Lipofectamine™ 2000 (Invitrogen) according to the manufacturer’s instructions. The supernatant containing virus source was collected 60 h post-transfection and filtered by a filter with 0.45-μm pore size (Millipore).

HCCC-9810 and CCLP1 cells were grown in log phase and then transfected with either pCDH-CMV-EF1-copGFP (vector) or PCDH-miR-144 for 12 h. Stable cell lines were screened by mass sorting on a FACSAria flow cytometer (BD Biosciences, Mountain View, CA, USA) based on the expression of GFP carried by the lentviral vector 72 h after transfection.

### RNA extraction and q-PCR

Total RNA was extracted from CCA cancer tissues or cells using Trizol reagent (Invitrogen) following the manufacturer’s instruction. For miR-144 detection, TaqMan miRNA expression assays were used to evaluate the expression of miR-144 using the StepOnePlus™ system (Applied Biosystems). To quantify the LIS1 mRNA levels, 500 ng of total RNA was subjected to first-strand cDNA synthesis using a PrimeScript RT Reagent kit (Takara, Dalian, China) according to the manufacturer’s instructions. q-PCR was performed with 2 × SYBR Green PCR master mix (Takara) on the iQ5™ quantitative PCR detection system (Bio-Rad, Richmond, CA, USA) and the results were analyzed with IQ5 software. The primers used in the reactions are listed in Additional file 1: Table S1. U6 and GAPDH were used as endogenous controls. All reactions were run in triplicate and all experiments were run in triplicate.

### Cell proliferation assays

Logarithmic growth phase cells were seeded at a density of 4 × 10^3^ cells per well in a 96-well plate containing 0.1 ml RPMI 1640 medium and 10% FBS. Cell Counting Kit-8 (CCK-8) (Dojindo, Tokyo, Japan) reagent was added at 0, 24, 48, and 72 h after seeding and incubated at 37°C for 2 h. The data of OD (optical density) value at 450 nm were measured by a microplate reader (Bio-Rad). Each experiment was performed three times with five replicates.

### Cell migration and invasion assays

Migration and invasion activities of cells were evaluated by wound healing and invasion assays. Cells were seeded in 6-well plates and the confluent monolayer cells (at 80% confluence) were scratched with a sterile 100-μL pipette tip. Images of the migrated cells were taken using a digital camera (Leica, Heerburg, Germany) 48 h later. The extent of wound healing was assessed by the distance traversed by cells migrating into the denuded area. In the invasion assay, the upper chamber of the transwell inserts with 6.5-mm polycarbonate membranes with 8.0-μm pores (Corning) was coated with Matrigel mixed with serum-free medium (diluted at 1:5) (BD Biosciences). A total of 5 × 10^4^ cells were resuspended in serum-free medium and placed in the upper chamber, while 600 μL medium containing 20% FBS was placed in the lower well. After 48 h, cells that did not migrate or invade were removed using a cotton swab. Invasive cells at the bottom of the membrane were washed twice with PBS, fixed in 4% paraformaldehyde, stained with 0.1% crystal violet, and counted under an inverted microscope. All experiments were performed in triplicate and were repeated three times.

### Luciferase reporter assay

HEK293T cells at 50% confluence in 96-well plates were co-transfected with hsa-miR-144 or anti-miR-144, along with reporter vectors using Lipofectamine™ 2000. The firefly and Renilla luciferase activities were measured 48 h after transfection using the Dual-Luciferase Reporter Assay System (Promega) on an illuminometer (Lumat LB 9507, Berthold, Germany). Renilla luciferase acted as a reporter gene and firefly luciferase as a normalized control for each individual analysis.

### Tumorigenicity assays in nude mice

The use of nude mice complied with the NIH Guide for the Care and Use of Laboratory Animals and local institutional ethical guidelines, and the study was approved by the Experimental Animal Ethics Committee of Tongji Medical College of Huazhong University of Science and Technology. Eight mice were used in each group. Four-week-old female nude mice (BALB/c-nude) were injected subcutaneously in the upper back with 5 × 10^6^ CCLP1 cells stably transfected with empty vector or miR-144 vector in 150 μL sterile PBS. Tumor growth was measured with calipers every 7 days and the tumor volumes were calculated using the formula: 1/2 (length × width^2^). Mice were sacrificed and tumor weight was examined 5 weeks later.

### Western blot

Cells were harvested with 1× cell lysis buffer (Promega). A total of 60 μg of total proteins were separated on 10% polyacrylamide gel and transferred to nitrocellulose membranes (Bio-Rad). The membranes were blocked with 1% bovine serum albumin in TBST buffer (Tris Buffer Saline containing 0.1% Tween-20) for 1 h at room temperature, and subsequently incubated with antibodies against LIS1 (PA5-20419, pierce), Akt (#4691, Cell Signaling Technology), p-Akt (#4058, Cell Signaling Technology), MMP-2 (#4022, Cell Signaling Technology), or GAPDH (#2118, Cell Signaling Technology) overnight at 4°C. After extensive washing with TBST buffer, the blots were then incubated with goat anti-rabbit, horseradish peroxidase-conjugated secondary antibody for 1 h at room temperature. Protein bands were detected by enhanced chemiluminescence reagents ECL (Millipore, MA, USA) and the intensity of the bands was analyzed using Image J software (National Institute of Health, USA).

### Statistical analysis

Data were expressed as the mean ± standard deviation (SD). Statistical analyses were performed with GraphPad Prism 5.0 (GraphPad Software). miR-144 expression was compared in CCA tissues and matched adjacent, non-tumor bile duct tissues by the paired Student’s t test. The relationship between miR-144 and LIS1 expression was carried out by Spearman’s correlation. A *P*-value of <0.05 was regarded as statistically significant.

## Results

### The miR-144 is downregulated in CCA tissues and cell lines

**Figure 1:**
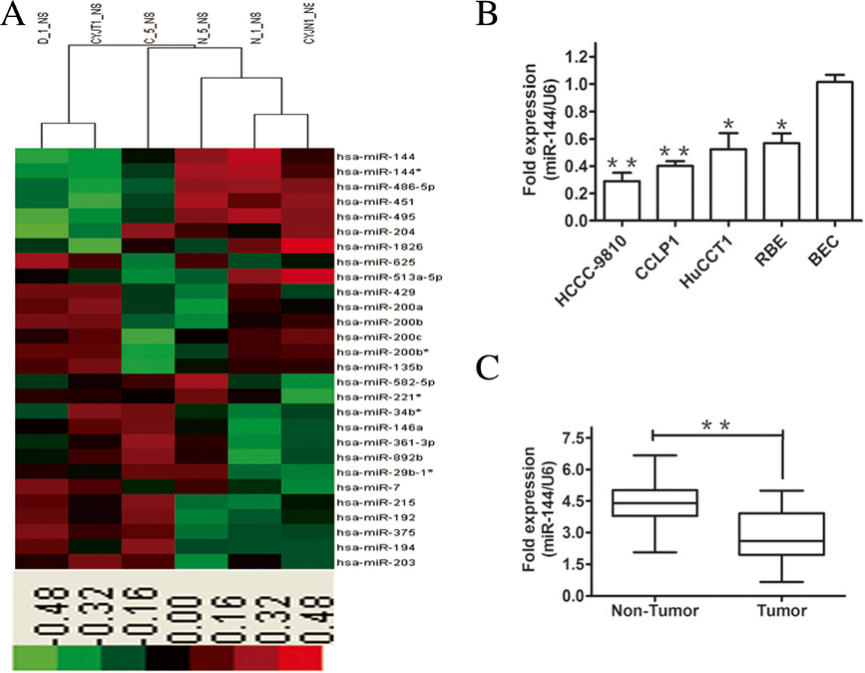
**Differential miRNA expression profiles in malignant bile ducts and adjacent normal bile ducts. A**. Unsupervised hierarchical clustering was performed using log2 relative frequencies of 29 differentially expressed miRNAs. Red represents overexpressed miRNAs; green represents underexpressed miRNAs. **B**. Endogenous levels of miR-144 in HCCC-9810, CCLP1, HuCC-T1, RBE, and BEC cells detected by q-PCR analysis. **C**. Relative expression of miR-144 in CCA tissues was significantly lower than that in corresponding normal bile duct tissues. Tumor indicates CCA tissue samples, and non-tumor indicates adjacent normal bile ducts. U6 was used as a loading control. *P < 0.05, **P < 0.01.

As miRNAs have critical functions in the regulation of many biological processes such as cell proliferation and metastasis, we compared the miRNA expression profiles between three pairs of CCA samples and the corresponding normal bile duct tissues. Taking a twofold difference as the cut-off point, microarray analysis detected 19 upregulated and 9 downregulated miRNAs in the three pairs of CCA and corresponding non-tumor tissues (Figure 1A). To study the possible role of miRNAs that inhibit CCA cell proliferation and metastasis, we focused on miR-144, as it is significantly downregulated in CCA samples. To validate the expression of miR-144 in miRNA microarray data, we confirmed that the expression of miR-144 was remarkably reduced in the three CCA samples compared with the corresponding normal bile duct tissues (data not shown).

Next, we compared the expression of miR-144 in a panel of human CCA cell lines (HCCC-9810, CCLP1, HuCCT1, and RBE) and the non-malignant cell line BEC, and the results showed that miR-144 expression was significantly decreased in these CCA cell lines (*P* <0.05) (Figure 1B). Furthermore, we measured the expression levels of miR-144 in the 70 pairs of human primary CCA tumor and adjacent, normal bile duct tissue samples and found that miR-144 was significantly downregulated in CCA cancer tissues compared with adjacent non-neoplastic tissues (*P* <0.01) (Figure 1C). These data indicated that miR-144 was decreased in CCA cancer tissues and cells, and thus it might be involved in human CCA development.

### MiR-144 suppresses cell proliferation, migration and invasion in CCA cells

**Figure 2:**
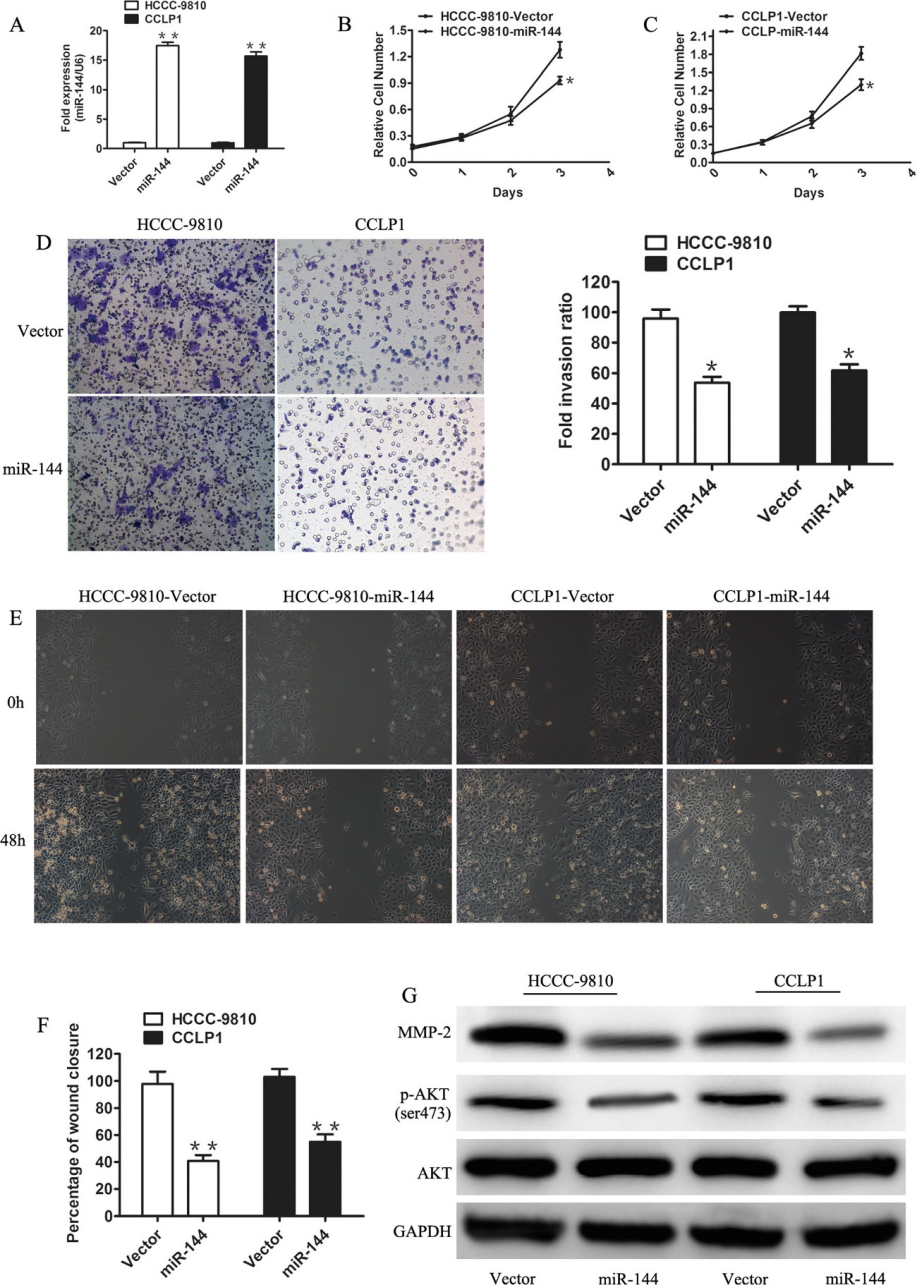
**MiR-144 suppressed proliferation and invasion of cholangiocarcinoma cell lines through the AKT Pathway. A**. Relative miR-144 expression level in HCCC-9810 and CCLP1 cells transfected with the control (empty vector) or miR-144-expressing vector (miR-144). **B** and **C**. Effects of stably expressing miR-144 on CCA cell proliferation were determined by cell proliferation assay. **D**. Representative images of invasion assays. The bar graph showed quantification of cell invasion between the miR-144-transfected cells and the control cells. **E**. Representative results of wound healing assays. **F**. Quantification of migrated cells with indicated treatment. **G**. Western blot analysis to assess the protein levels of AKT, p-AKT, and MMP2 in HCCC-9810 and CCLP1 cells with miR-144 overexpression. GAPDH was used as an internal control to normalize protein signal intensity. Data are shown as mean ± SD and were representative of three independent experiments. *P <0.05, **P < 0.01.

To investigate the effects of miR-144 in CCA cells, HCCC-9810 and CCLP1 cells were transfected with either vector or pCDH-miR-144. We confirmed that miR-144 was significantly increased in HCCC-9810 and CCLP1 cells after transfection with pCDH-miR-144 compared with the vector by qPCR (*P* <0.01) (Figure 2A). Overexpression of miR-144significantly decreased cell proliferation compared with control cells expressing vector after 48 h and 72 h (*P* <0.05) (Figure 2B, C).

The vast majority of deaths from cancer are due to tumor cells spreading from the primary tumor to other parts of the body. We therefore investigated whether reintroducing miR-144 would decrease the invasive and migration potential of CCA cells. Transwell assay was performed to analyze the effect of miR-144 on the invasive behavior of HCCC-9810 and CCLP1 cells. Overexpression of miR-144 reduced cell invasion abilities of the CCA cells compared with the control cells (Figure 2D). Quantitative analyses of the results indicated that migration of miR-144-overexpressed cells was decreased at 48 h compared with control cells (*P* <0.05) (Figure 2D). Scratch assays showed that miR-144-overexpressed cells retained a larger scratch area (*P* <0.05) (Figure 2E,F).

It is well known that the Akt signaling pathway is the most important survival pathway that plays a central role in diverse cellular functions, including proliferation and metastasis and the phosphorylation of AKT atS473 is the best marker of activated AKT signaling pathway. So after we saw the inhibition effect of miR-144 on CCA cell proliferation, the first thing we thought about was to detect the status of pAKT (S473). The data showed that the level of p-AKT was significantly decreased in cells overexpressing miR-144 compared with control cells (Figure 2G). Moreover, it was reported that tumor cell invasion is activated by MMP2, which is a 72 kDa type IV collagenase involved in the breakdown of extracellular matrix to help cell invasion [23], and AKT signaling pathway activates MMP2 and enhances cell invasion in different cancers, such as breast cancer and lung cancer [24, 25]. As predicted, miR-144 overexpression reduced the expression of the MMP2 which explained why the cell incvasion activity decreased in both HCCC-9810 and CCLP1 cells (Figure 2G). These observations indicated that miR-144 inhibits cell growth and invasion through suppression of AKT signaling pathway.

### LIS1 is a direct target of miR-144

**Figure 3:**
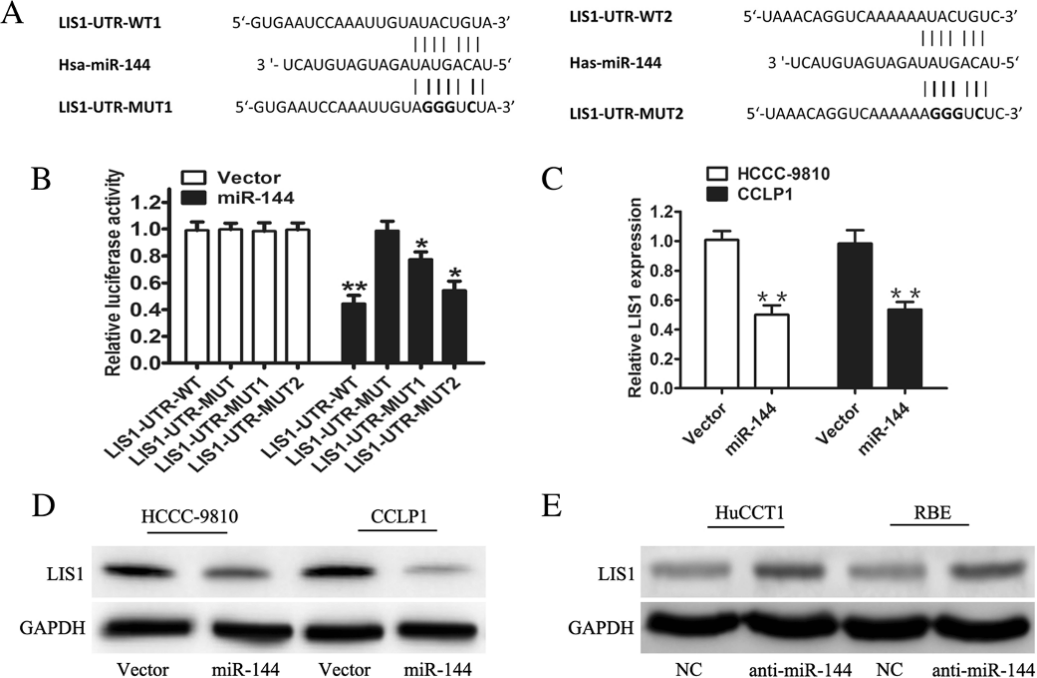
**LIS1 is a new target regulated by miR-144. A**. Schematic of the two predicted seed regions in the 3′-UTR of LIS1 and mutated 3′-UTR. **B**. Luciferase activity was assayed in HEK293T cells. The wild-type (WT) or mutated LIS1 3′-UTR reporter gene vector was co-transfected with vector or miR-144 (MUT1 and MUT2: mutations of each binding site; MUT: mutation of both binding sites). All values represent the mean of five independent experiments performed in duplicate. **C**. q-PCR analysis examined the mRNA level of LIS1 after overexpression of miR-144 in HCCC-9810 and CCLP1 cells. **D**. Endogenous protein levels of LIS1 in HCCC-9810 and CCLP1 cells after indicated treatments were detected by western blots. **E**. Western blot showed the expression of LIS1 in HuCTT1 and RBE cells transfected with negative control oligonucleotide (NC) or miR-144 inhibitor (anti- miR-144). Data are shown as mean ± SD and were representative of three independent experiments. *P <0.05, **P < 0.01.

There is no evidence that miR-144 suppresses AKT activity directly. To explore the underlying mechanisms by which miR-144 inhibits the proliferation and invasion of CCA cells through suppression of AKT, the direct target genes of miR-144 were obtained from public databases. LIS1 was one of the predicted targets, and we examined the 3′-UTR of LIS1 mRNA and identified two conserved putative miR-144 binding sites by computational algorithms (Figure 3A). To verify LIS1 as a direct target of miR-144, we co-transfected HEK293T cells with a LIS1 luciferase reporter vector which contains a wild-type or mutated binding site for miR-144 together with or without miR-144, followed by luciferase reporter assay. To further examine the relative contribution of the two candidate miR-144-binding sites, mutations of four nucleotides in each seed-binding site (MUT1 and MUT2) and a mutation of two binding sites (MUT) were included, which abolish the putative miRNA-mRNA interactions (Figure 3A). Our data indicated that miR-144 attenuated the luciferase activity of the wild-type 3′-UTR of LIS1 (*P* <0.01) and reduced the luciferase activity of both the individual binding site mutants (*P* <0.05) (Figure 3B), but the activity of the double mutant 3′-UTR vector remained unaffected. These results showed that both putative miR-144-binding sites contribute to the regulation of LIS1. In addition, overexpression of miR-144 resulted in decreased endogenous mRNA and protein levels of LIS1 in CCA cells compared with cells transfected with the control vector (Figure 3C and D). On the contrary, western analysis showed that LIS1 protein expression was significantly increased in miR-144 inhibitor transfected cells (Figure 3). Taken together, these results demonstrated that miR-144 inhibited the expression of LIS1 through post-transcriptional repression.

### LIS1 is a functional target of miR-144 and is inversely correlated with miR-144 levels

**Figure 4:**
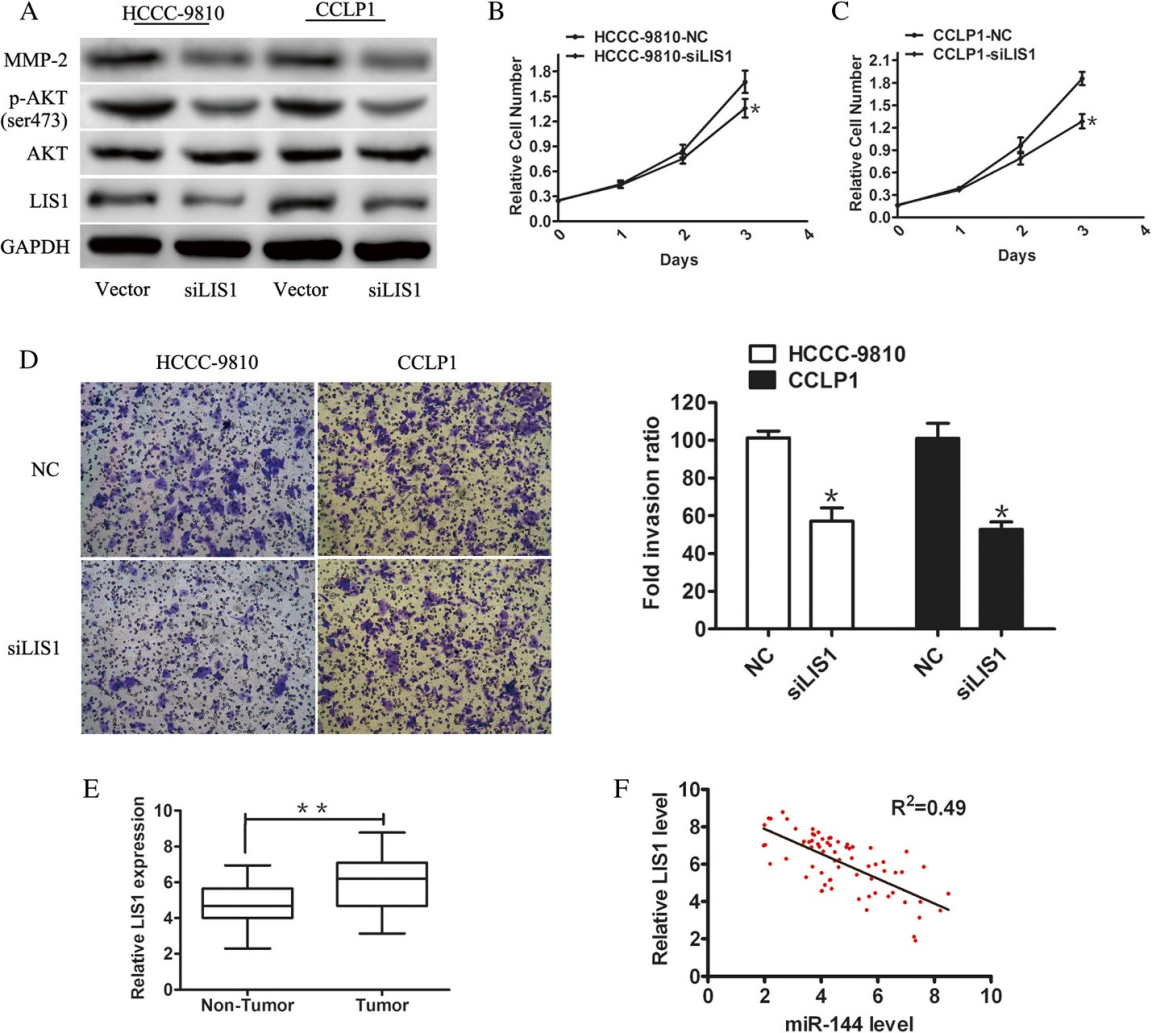
**Effects of siRNA-mediated knockdown of LIS1 on CCA cell proliferation, invasion, and migration. A**. Western blot was used to detect the expression of LIS1, AKT, p-AKT, and MMP2 in HCCC-9810 and CCLP1 cells transfected with indicated siRNAs (20 nmol/L) 48 h after transfection. **B** and **C**. The cell proliferation curve of HCCC-9810 and CCLP1 cells transfected with LIS1 siRNA or control for 48 h. **D**. Representative results of invasion assays. The bar graph showed quantification of cell invasion between cells transfected with LIS1 siRNA and control. **E**. The mRNA levels of LIS1 were detected in CCA tumor tissues or matched adjacent tissue by q-PCR.GAPDH was used as a internal control. **F**. Expression of miR-144 was inversely correlated with LIS1 in CCA tissues. Statistical analysis was performed using Pearson’s correlation coefficient. (R^2^ = 0.49, *P* <0.01). *P < 0.05, **P <0.01.

To investigate the effects triggered by the targeting of LIS1 by miR-144 in cultured CCA cells, we used small interfering RNA (siRNA) knockdown of LIS1 to evaluate effects in proliferation and invasion. Transfection of si-LIS1 resulted in efficient depletion of LIS1in both HCCC-9810 and CCLP1 cells compared with cells transfected with negative control (NC) (Figure 4A). The levels of p-AKT, and MMP-2 also decreased (Figure 4A). Cell proliferation assays revealed that knockdown of LIS1 suppressed proliferation of HCCC-9810 and CCLP1 cells (*P* <0.05) (Figure 4B,C). In addition, LIS1 silencing reduced capabilities of cell invasion both in HCCC-9810 and CCLP1 cells compared with control cells (*P* <0.05) (Figure 4D).

Prior evidence suggested that LIS1 was upregulated in lung cancer patients and enhanced migration and invasion of lung cancer cells [26]. To investigate the expression of LIS1 in CCA patients, we examined the mRNA level of LIS1 in the same patient cohort used for measuring miR-144 levels and found an increased expression of LIS1 mRNA levels in tumors compared with normal controls (*P* <0.01) (Figure 4E). Of interest, an inverse correlation was observed between miR-144 and LIS1 in human CCA tissues (Figure 4F). These results confirmed LIS1 as a functional downstream target of miR-144.

### MiR-144 attenuated the growth of CCA cells in a nude mouse xenograft model

**Figure 5:**
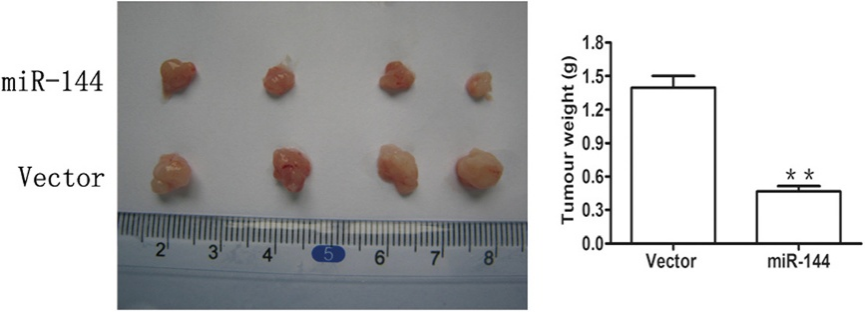
**miR-144 suppresses tumor growth** ***in vivo*** **.** Representative tumor xenografts excised from SCID mice. Quantitation of tumor weight in recipient mice at week 5 after indicated treatments. **P < 0.01.

Our previous studies showed that miR-144 restoration inhibited cell growth and invasiveness *in vitro*. Therefore, we performed xenograft formation assays to evaluate the effect of miR-144 overexpression on tumorigenicity. Stable transfection of miR-144 in CCLP1 cells delayed tumor formation and caused a dramatic reduction of tumor size compared with the control vector (Figure 5A). Analysis of tumor weight revealed that miR-144 overexpression markedly reduced tumor growth after 5 weeks compared with vector tumors (*P* <0.01) (Figure 5B). All these findings further indicated the tumor suppressive effect of miR-144 on CCA both *in vitro* and *in vivo*.

## Discussion

CCA is the most malignant neoplasm compared with many other malignant tumors [3, 4, 7, 8]. Although surgical resection plus adjuvant chemotherapy results in better outcome of early-stage patients, long-term survival remains low. Accumulating evidence shows that expression levels of various miRNAs are downregulated in cancer, indicating that these miRNAs may act as tumor suppressors [27–29]. However, the roles of miRNA in cell proliferation and invasion of CCA are not yet fully clear. In this study, we used miRNA expression profiles to identify 28 differentially expressed miRNAs. This approach confirmed the differential expression of several miRNAs such as miR-495 and miR-486, which are involved in cancer cell growth and invasion *in vitro* and *in vivo* [30, 31].

Although the research on the true biological relevance of miR-144 in cancer is still in its infancy, in this study, we focused on the expression and function of miR-144 in CCA. MiR-144 has been implicated in both oncogenic or tumor suppressor roles in different tumors [32–34], but its role in CCA is not yet clarified. Here, we found that miR-144 acts as a possible tumor suppressor in CCA. First, our data show that miR-144 is significantly downregulated in CCA tissues and cell lines. Furthermore, we found that forced expression of miR-144 could significantly attenuate cell proliferation, migration, and invasion *in vitro*. The inhibitory effect of miR-144 was also confirmed in CCA cells *in vivo*.

To elucidate the mechanism of miR-144 inhibiting cancer, first, we tested the AKT signaling pathway, which is usually activated in cancer and increase cell growth. We saw activity of AKT was decreased significantly by overexpression of miR-144. Furthermore, MMP2, a collagenase which promotes cell invasion and is activated by AKT, was downregulated by miR-144. All these showed that miR-144 inhibits CCA cell growth and invasion through suppressing AKT. But we could not find any direct regulation of AKT by miRNAs. Then we tried to find the direct target of miR-144, which would explain how miR-144 suppresse AKT. Platelet-activating factor acetylhydrolase 1b, also named LIS1, was first identified as a causative gene for type I lissencephaly disease. Here, we predicted that LIS1 is a potential target of miR-144 based on the evolutionally conserved 3′-UTR sequence by bioinformatics analysis and we also proved that it is a direct target of miR-144. A previous study indicated that LIS1 could organize the microtubule cytoskeleton and regulate cell migration in Drosophila [35]. A recent study reported that LIS1 overexpression promoted cancer cell invasion and was significantly associated with poor survival in lung adenocarcinoma [26]. Our results revealed that the expression of LIS1 is remarkably increased in CCA tissues, and knockdown of LIS1 by siRNA has similar inhibition effect on the growth and motility properties of CCA cell lines with miR-144 overexpression. In another word, even there is other direct target of miR-144, the inhibition effect of miR-144 in CCA is mainly through decreasing LIS1. However, some studies showed that LIS1 was downregulated in hepatocellular carcinoma and ectopic expression of LIS1 could significantly inhibit hepatocellular carcinoma cell proliferation and colony formation [36]. The discrepancy of LIS1 expression between CCA and hepatocellular carcinoma might be due to tissue-specific gene expression and functions. The decrease of p-AKT was also observed when knocking down LIS1 by siRNA. It suggests that the regulation of LIS1 by miR-144 is in the upstream of AKT and LIS1 may be a upstream activator of AKT directly or undirectly.

This study presents that miR-144 is a tumor suppressor in CCA through inhibition of AKT and also finds its direct target LIS1, which is the major effector in the tumor suppression process. Besides, a new direction is addressed by this study that LIS1 could affect AKT signaling pathway.

## Conclusions

Here, we conducted miRNA expression profiling analysis between the CCA tissues and paired bile ducts, followed by experimental validation in CCA tissues and cell lines. We found that miR-144 is down-regulated in CCA, and our results suggest that miR-144 inhibits CCA cell growth and invasion through decreasing p-AKT and directly targeting LIS1. Thus, these studies not only substantially broaden our understanding of the complex mechanisms underlying the pathogenesis of CCA but also implicate potential new interventional and therapeutic strategies. However, future studies examining the correlation between miR-144 expression status and clinic pathological parameters and elucidating the precise signal transduction pathways of miR-144 are warranted.

## Electronic supplementary material

Additional file 1: Table S1: Nucleotide sequences in our research. (DOC 32 KB)
