# Supplementary material for: MicroRNA-144 suppresses cholangiocarcinoma cell proliferation and invasion through targeting platelet activating factor acetylhydrolase isoform 1b
Source: BMC Cancer. 2014 Dec 5;14:917. doi: 10.1186/1471-2407-14-917 (PMC4289222; doi:10.1186/1471-2407-14-917)
Supplement: Supplementary file 1 — Additional file 1: Table S1: Nucleotide sequences in our research. (DOC 32 KB) [file 12885_2014_5093_MOESM1_ESM.doc]

| Additional_file 1:Figure S1. Nucleotide sequences in research | |  |
| --- | --- | --- |
| Name | Forward primer  sequence (5'-3') | Reverse primer  sequence (5'-3') |
| Primers for Gene or 3'UTR Cloning | | |
| PCDH-miR-144 | AAAGAATTCGAGCAGAGAGCTTCTT  GGGC | AAAGGATCCTCCAGCCCTGA  CCTGTCCT |
| Psi-check2-LIS1 3'UTR | AAACTCGAGTTGTGTCTCCTTCGG  CCC | AAAGCGGCCGCGGCATTTA  ATAGTTTACCAGTTGGT |
| siRNA Duplexes | | |
| LIS1 siRNA | UGACCAUUAAACUAUGGGAUU |  |
| Control siRNA | AACGUACGCGGAAUACUUCGA |  |
| Primers for RT-PCR | | |
| LIS1 | GACTGTGCGTGTATGGGTCG | AGGGTGCCGTCTTGTGGA |
| GAPDH | ATCCCATCACCATCTTCCAG | CCATCACGCCACAGTTTCC |
